# Two Cases of Hepatic Epithelioid Hemangioendothelioma Misdiagnosed With Hepatic Veno‐Occlusive Disease

**DOI:** 10.1155/crhe/8493924

**Published:** 2026-01-31

**Authors:** Bing Zhu, Yiwen Xv, Fangjiao Song, Sa Lv, Jinghui Dong, Shuhong Liu, Shaoli You

**Affiliations:** ^1^ Senior Department of Hepatology, Chinese PLA General Hospital, Beijing, China, 301hospital.com.cn; ^2^ Senior Department of Radiology, Chinese PLA General Hospital, Beijing, China, 301hospital.com.cn; ^3^ Senior Department of Pathology, Chinese PLA General Hospital, Beijing, China, 301hospital.com.cn

**Keywords:** hemangioendothelioma, liver biopsies, veno-occlusive disease

## Abstract

Hepatic epithelioid hemangioendothelioma (HEHE) is a rare tumor of vascular origin with an incidence of < 0.1/100,000. The disease is easily misdiagnosed. The aim of this article is to increase public awareness and vigilance of HEHE. We report two cases presenting with fever, abdominal discomfort, abnormal liver function, and jaundice. Computed tomography (CT) and magnetic resonance imaging (MRI) of the abdomen suggested hepatic stasis, venous compression, and ascites, and liver biopsy was initially misdiagnosed as hepatic veno‐occlusive disease (VOD), which did not improve with treatment. In the first case, a male patient, after liver transplantation, pathological and immunohistochemical (IHC) analyses revealed hyperplasia of blood vessels in the liver tissue with dilated lumen and heterogeneous cells. Immunohistochemistry was performed and showed CD34 positivity, confirming the diagnosis of HEHE. The second female patient had liver bruising and ascites on imaging, and the first hepatic puncture was reported to be VOD, which did not improve with treatment. Repeat hepatic puncture was performed, and the diagnosis of HEHE was confirmed after a second repathology with additional immunohistochemistry for HEHE. These misdiagnosis cases highlight the challenge of diagnosing HEHE. This is the first report of misdiagnosis of HEHE as VOD. This article analyzes the underlying causes of misdiagnosis of HEHE and emphasizes the causes of imaging misdiagnosis and the importance of repeated hepatic puncture biopsy and immunohistochemistry in the diagnosis of HEHE.

## 1. Introduction

Hepatic epithelioid hemangioendothelioma (HEHE) was first described and named by Weiss and Enzinger in 1982 [1] and was confirmed to be an intermediate vascular tumor. In 2002, the World Health Organization’s (WHO) pathological and genetic classification of soft tissue and bone tumors categorized it as malignant hemangiomas [2]. Due to the absence of distinctive imaging features, HEHE shares clinical similarities with hepatic veno‐occlusive disease (VOD), Budd–Chiari syndrome, and cirrhosis, often resulting in misdiagnosis.

## 2. Case Presentation

### 2.1. Case 1

**FIGURE 1:**
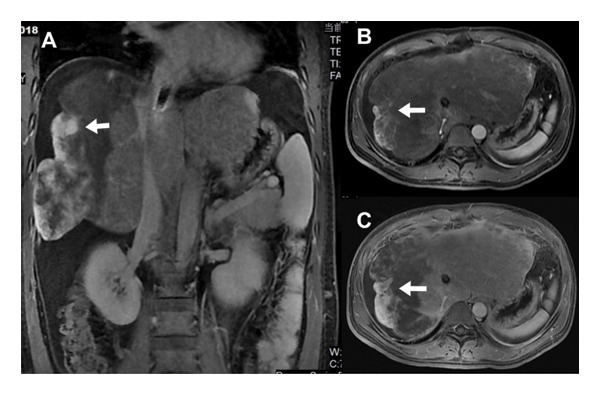
MRI imaging of Case 1: (A) coronal‐enhanced scan in the delayed phase reveals inhomogeneous enhancement of intrahepatic masses, accompanied by perihepatic ascites. (B) Axial T1WI arterial phase scan displays diffusely reduced enhancement of intrahepatic mass shadows. (C) Axial delayed phase scan exhibits contrast agent filling from the margin to the center of the mass in the right lobe of the liver).

**FIGURE 2:**
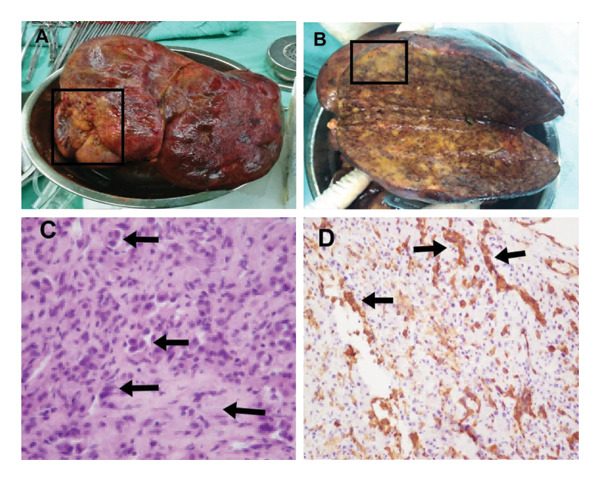
Hepatic histopathology of Case 1: ((A) macroscopic specimen of transplanted diseased liver. (B) Diseased liver as observed with the naked eye upon incision. (C) Tumor cells identified through HE staining of liver tissue, × 200; (D) strongly positive CD34 staining of liver tissue).

A 37‐year‐old male was diagnosed with abnormal liver function in December 2018. By April 2019, he experienced intermittent fever, peaking at 38.0°C. Examination revealed his liver extended 3 cm below the right rib and had a medium texture. Murphy’s sign was negative. The spleen was not palpable beneath the left subcostal area, with no lower limb edema noted. Initial laboratory tests indicated ALT/AST 79/63 U/L, T/DBIL 24/6 μmol/L, PTA 69%, CA199 66.47 U/mL, CA125 1584 U/mL, negative screenings for Hepatitis B and C, cytomegalovirus, Epstein–Barr virus, normal ceruloplasmin levels, and negative autoantibodies and Hepatitis A and E antibodies. Abdominal computed tomography (CT) suggested heterogeneous liver densities, bruising manifestations, hepatic venous compression with enhancing nodules, splenomegaly, and ascites, and the diagnosis of Budd–Chiari syndrome or VOD was considered. Gastroscopy revealed mild esophageal varices and chronic superficial gastritis with erosions. After ineffective drug combination therapy, the patient underwent transjugular intrahepatic portosystemic shunt (TIPS) with transjugular vein hepatic puncture in June, and the pathology report suggested the diagnosis of VOD. Postoperative liver failure occurred, and after treatment proved ineffective, the patient underwent a liver transplant in July. Post‐transplant pathology identified diffuse vascular proliferation, dilated vascular lumens, papillary projections of atypical cells, scattered tumor cell clusters forming small lumens, marked cellular atypia, intracytoplasmic cavity‐like structures, and interstitial mucus degeneration, affecting multiple liver lobes, some aligning with veins and hepatic sinusoids (Figures 1 and 2). Immunohistochemistry confirmed CD34(+) positivity without cirrhosis, leading to a diagnosis of HEHE. The patient died 4 months after liver transplantation due to diffuse intrahepatic recurrence of the tumor.

### 2.2. Case 2

**FIGURE 3:**
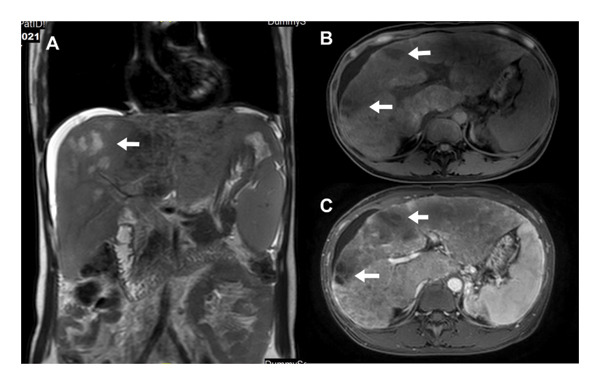
MRI image of Case 2: (A) coronal T2‐weighted image displaying a diffusely inhomogeneous signal in the liver parenchyma, visible ascites surrounding the liver, and multiple patchy high‐signal shadows within the mass in the right anterior lobe. (B) Axial T1‐weighted image showing low‐signal mass shadows in the left medial and right anterior lobes of the liver. (C) Axial‐enhanced scan during the portal venous phase indicating mass‐like reduced enhancement of tumor shadows).

**FIGURE 4:**
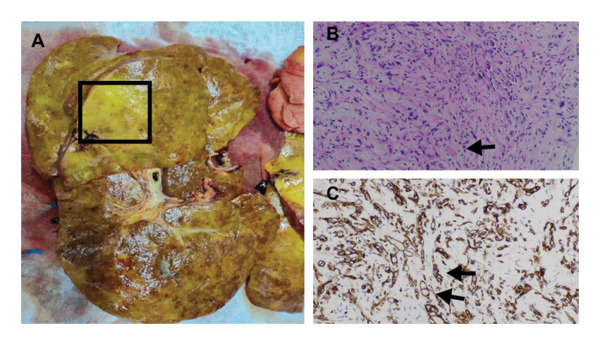
Liver histomorphology of Case 2: ((A) gross examination of the transplanted liver with an open incision. (B) Histological examination showing tumor cells at 200x magnification with HE staining. (C) Liver tissue displaying strong positivity with CD34 staining).

A 39‐year‐old female, diagnosed as HBsAg positive during a pregnancy test in 2004, presented with normal liver function and received no treatment. In February 2021, she experienced intermittent epigastric discomfort without clear triggers, which gradually worsened. This was accompanied by intermittent fever, peaking at 38.3°C, but without chills or shivering. The examination revealed liver enlargement, reaching 8 cm below the raphe and 4 cm below the right rib, with a hard texture and mild tenderness. Murphy’s sign was negative. The spleen was palpable 2 cm below the left rib. Liver tests revealed ALB 30 g/L, TBIL 20.8 umol/L, ALT 49.7 U/L, AST 54 U/L, ALP 134 U/L, GGT 42 U/L, CHE 2574 U/L, CA199 13.70 U/mL, CA 239.50 U/mL, and HBVDNA 2.08*E* + 02 IU/mL. Tests for Hepatitis A, C, E, and autoantibodies were negative, and neither bone marrow puncture nor ascites culture indicated any abnormalities. Abdominal CT showed heterogeneous liver density, bruising manifestations with enhancing nodules, and ascites. A transjugular vein hepatic puncture diagnosed VOD, hepatic fibrosis, and chronic viral Hepatitis B. Despite low molecular weight heparin anticoagulation therapy, the patient continued to suffer repeated fevers, and her condition did not improve with anti‐infection treatment. Her condition deteriorated by August 2021, prompting a visit to our hospital. Abdominal magnetic resonance imaging (MRI) indicated hepatic venous perfusion anomalies, intrahepatic arterial phase enhancement nodules, cirrhosis, splenomegaly, and ascites. Gastroscopy showed mild esophageal varices. Further lab tests showed elevated levels of total bilirubin, direct bilirubin, alkaline phosphatase, alanine aminotransferase, r‐glutamyl transferase, aspartate aminotransferase, and the international normalized ratio, while cholinesterase levels decreased. Due to the limited amount of original pathological tissue samples, a repeat liver biopsy was performed. The liver biopsy revealed fusiform blood vessels, and fibrous tissues were found in the liver tissue; the blood vessels were incompatible and lined with fat spindle cells, and red blood cells were found in the lumen. Many fibrous tissues around the blood vessels were accompanied by hyalinoid transformation and mixed bile ducts (Figures 3 and 4). Further immunohistochemical analysis confirmed the lesion’s characteristics as an epithelioid hemangioendothelioma, with positive staining for the CD34 antibody. On August 28, 2021, the patient underwent a liver transplant. The pathology of the diseased liver was confirmed as HEHE. The patient survived with a 2‐year follow‐up.

## 3. Discussion

HEHE, an infrequent vascular‐origin tumor, is moderately or low‐grade malignant. This disease’s incidence is low, and its etiology remains uncertain, possibly linked to sex hormones, vinyl chloride exposure, chronic Hepatitis B virus infection, and alcoholism [3, 4]. Clinical manifestations are variegated and include discomfort or pain in the right upper abdomen, weight loss, sporadic jaundice, fever, and fatigue [5]. Some patients display normal laboratory test results, yet exhibit elevated serum alkaline phosphatase activity and alanine aminotransferase levels. The atypical clinical presentations and absence of specific laboratory markers complicate HEHE detection and diagnosis, resulting in approximate misdiagnosis rates of 60%–80% initially [6]. In this report, both patients exhibited a range of common liver disease symptoms, which complicated the differential diagnosis. The misdiagnosis largely stemmed from two factors: First, imaging showed uneven liver density with compression of the hepatic veins and congestive features, accompanied by enhancement nodules; these findings resembled VOD or Budd–Chiari syndrome. Second, in both cases, the initial liver‐puncture pathology via the right internal jugular approach was reported as VOD.

HEHE often lacks distinctive imaging features. Ultrasound, CT, and MRI can detect hepatomegaly, splenomegaly, ascites, and portal hypertension. CT scans may reveal multiple, homogeneous low‐density lesions, with peripheral contrast enhancement and possible formation of a target sign or halo due to mucinous substances within the tumors [7]. On MRI, multiple peripheral hepatic lesions are commonly observed, particularly subcapsular lesions associated with capsular retraction. These lesions typically exhibit low signal intensity on T1‐weighted images (T1WI) and high‐signal intensity on T2‐weighted images (T2WI), with the lesion center appearing hyperintense. Dynamic contrast‐enhanced MRI shows marginal enhancement in the arterial phase, with progressive enhancement in the portal venous and delayed phases, resulting in a halo or target sign. Although the “lollipop sign” has been reported as a specific imaging feature of HEHE [8], the pathological underpinning of the “lollipop sign” is that the tumor cells encapsulate and invade the hepatic vein, portal vein, and its branches, resulting in stenosis or even occlusion of blood vessels, which is a typical manifestation of vascular invasion [9, 10]. The two patients discussed in this paper, these signs were similarly present in both cases but were atypical. Therefore, imaging studies alone cannot fully satisfy the diagnostic requirements for HEHE.

**FIGURE 5:**
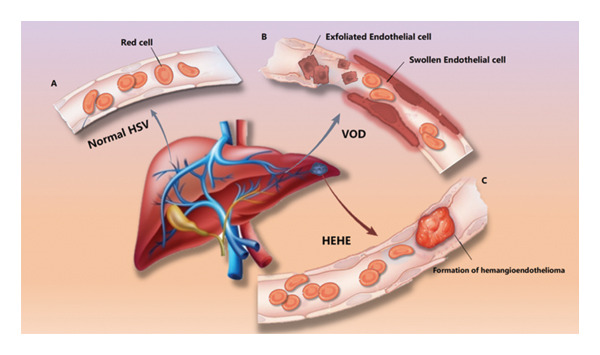
Schematic diagram of hepatic veno‐occlusive disease (VOD) misdiagnosis mechanism: (A) normal hepatic sinusoid blood flow. (B) In VOD, swelling and shedding of sinusoidal endothelial cells obstruct blood flow. (C) HEHE cells obstruct hepatic venules, further inhibiting blood flow. Similar mechanisms in (B) and (C) produce analogous imaging and pathological outcomes).

HEHE is a slow‐growing neoplasm that can form tumor thrombi, potentially occluding the hepatic artery or vein over time. As HEHE predominantly develops in the endothelium of small hepatic veins, it can lead to postsinusoidal portal hypertension and imaging characteristics of VOD [11], which may result in misdiagnosis, as depicted in Figure 5. Hepatic vein invasion may cause hepatic stasis, producing imaging features of Budd–Chiari syndrome and increasing the likelihood of misdiagnosing Budd–Chiari syndrome itself [12, 13]. The pathologic localization of HEHE manifests as hepatic venous obstruction, which is the cause of misdiagnosis in imaging studies. Therefore, when clinical imaging reveals features suggestive of VOD or Budd–Chiari syndrome, further differential diagnosis with HEHE is warranted.

Pathological examination is the only method for definitive diagnosis. The recognition of the real nature of the tumor depends upon awareness of the morphological spectrum of neoplastic endothelium. In numerous instances of hemangioendothelioma, extensive fibrosis alongside diffuse, sparse, and intermingled epithelioid cells within the blood sinus enhances the risk of misdiagnosing a puncture biopsy sample as benign fibrous tissue, consequently overlooking the epithelioid cells [14]. Thus, when feasible, wedge biopsy or repeated puncture biopsy of larger samples is advocated, although these procedures pose bleeding risks and are challenging to execute. In both reported cases, a transjugular liver biopsy was conducted due to liver failure, mitigating the bleeding risk [15], and the small sample size may have contributed to the misdiagnosis of the cases. IHC is a definitive diagnostic method for HEHE, but IHC for HEHE is not a routine pathology test. Further IHC workup is only performed when there is clinical suspicion or when pathologists, during HE staining evaluation, raise suspicion for HEHE. In Case 1, the patient underwent liver transplantation after liver failure rescue failed; only upon re‐review of the explanted liver and completion of the IHC was the initial misdiagnosis revealed. In Case 2, the first liver biopsy diagnosed VOD; after ineffective VOD‐directed treatment, the diagnosis was reconsidered, a repeat liver biopsy was performed, and IHC was completed to establish the correct diagnosis. Therefore, experienced clinicians and pathologists should be vigilant during HE staining slide review, and complete IHC workup constitutes another key step in preventing misdiagnosis.

HEHE is resistant to both chemotherapy and radiotherapy, and liver transplantation emerges as a viable alternative [16, 17]. The 1‐, 3‐, and 5‐year survival rates following liver transplantation for HEHE are 80%, 68%, and 64% [18]. The death of the first patient in this study following liver transplantation may be related to factors such as the severity of their condition, the impact of the TIPS procedure, and the prolonged period of misdiagnosis. The second patient achieved excellent therapeutic outcomes. For patients lacking access to transplantation, surgical resection remains the primary treatment [19], but in most cases the multicentricity of the lesions makes complete local resection impossible. HEHE typically presents as multifocal lesions that are challenging to remove surgically, exhibit high rates of distant metastasis, and show poor response to treatments such as radiotherapy and chemotherapy [20]. Hepatic artery embolization therapy and ablation therapy may offer survival advantages for HEHE patients who are not candidates for surgery [21]. Thalidomide, known for its vascular endothelial inhibitory effect, may be beneficial in treating diffuse metastatic HEHE. The prognosis is largely influenced by the presence of extrahepatic metastasis at diagnosis, with the peritoneum, bones, and lymph nodes being the most frequent metastatic sites. Concurrent clinical symptoms or elevated CA199 levels might indicate a poor prognosis.

## 4. Conclusion

In summary, although hepatic vein obstruction exhibits typical radiological features, its etiology may involve multiple factors, necessitating careful differential diagnosis. HEHE may exhibit pathological features of hepatic venous obstruction, leading to misdiagnosis as VOD or BCS on imaging. Additionally, when performing pathological testing on such patients, ensure comprehensive immunohistochemical testing for HEHE. Repeated liver biopsies may be necessary to avoid misdiagnosis due to insufficient liver tissue sample volume.

## Funding

This work was supported by New Technologies and New Business Projects within the PLA General Hospital (grant number 2024‐XJS‐006) and National Science and Technology Major Projects (grant number 2025ZD01906302).

## Disclosure

All figures submitted have been created by the authors who confirm that the images are original with no duplication and have not been previously published in whole or in part.

## Consent

Written informed consent was obtained from the individual(s) for the publication of any potentially identifiable images or data included in this article.

## Conflicts of Interest

The authors declare no conflicts of interest.

## Data Availability

The data that support the findings of this study are available from the corresponding author upon reasonable request.
